# Impact of Decentralized Care and the Xpert MTB/RIF Test on Rifampicin-Resistant Tuberculosis Treatment Initiation in Khayelitsha, South Africa

**DOI:** 10.1093/ofid/ofv014

**Published:** 2015-02-04

**Authors:** Helen S. Cox, Johnny F. Daniels, Odelia Muller, Mark P. Nicol, Vivian Cox, Gilles van Cutsem, Sizulu Moyo, Virginia De Azevedo, Jennifer Hughes

**Affiliations:** 1 Division of Medical Microbiology and Institute of Infectious Disease and Molecular Medicine, University of Cape Town; 2 Médecins Sans Frontières; 3 National Health Laboratory Service; 4 City of Cape Town Health Department, Khayelitsha, South Africa

**Keywords:** delay, MDR-TB, RR-TB, treatment, Xpert

## Abstract

***Background.*** Globally, case detection and treatment access are poor for rifampicin-resistant tuberculosis (RR-TB). The Xpert MTB/RIF test has the potential to increase detection and reduce time to treatment (TTT). However, these benefits are dependent on health system capacity to provide treatment.

***Methods.*** We retrospectively assessed the impact of Xpert on treatment initiation and TTT in the context of decentralized RR-TB care in Khayelitsha, Cape Town, using routine programmatic data. Community-based treatment was introduced progressively from 2008. Before 2007, diagnosis relied on phenotypic resistance (culture). During 2007–2008, the line probe assay (LPA) was introduced, followed by Xpert in 2012.

***Results.*** Before decentralization (2003–2006), median TTT was 71 days (interquartile range [IQR], 49–134; *n* = 158). The LPA introduction during 2007–2008 was associated with reduced median TTT from 76 to 50 days (*P* < .0001, *n* = 257). Between January 2009 and June 2013, 938 RR-TB cases were diagnosed (74% human immunodeficiency virus [HIV]-infected). Decentralization during 2008–2011 was associated with declining TTT (*P* < .0001, test for trend), a decline to 28 days in 2011 (IQR, 16–40; *n* = 173). Xpert was associated with a further reduction to 8 days in 2013 (IQR, 5–25; *n* = 89; *P* < .0001). Treatment initiation remained unchanged with Xpert and was lower among HIV-infected (2010–2013); 87.9% (445 of 506) compared with 96.9% (188 of 194) for HIV-uninfected (*P* < .0001) patients.

***Conclusions.*** Improved case detection and rapid treatment initiation are required to interrupt transmission and reduce mortality. In this setting, decentralization was associated with high treatment initiation and reduced TTT. Xpert implementation significantly enhanced the reduction in TTT and has the potential to reduce transmission.

## Abstract (precis)

Decentralization of treatment for rifampicin-resistant tuberculosis was associated with high treatment initiation and resulted in reduced time to treatment initiation. Xpert for TB diagnosis resulted in a significant further reduction in time to treatment.

Direct transmission of drug-resistant tuberculosis (TB) is driving the epidemic in many high-burden settings [1, 2]. Therefore, high case detection, rapid diagnosis, and early treatment initiation are fundamental to reducing ongoing transmission [3]. As yet, however, case notification and access to treatment with second-line TB drugs remain poor. In 2013, only 20% of estimated multidrug-resistant TB (MDR-TB) cases were initiated on appropriate treatment globally [4], often with prolonged delays between a diagnostic sample and initiation of treatment, resulting in high mortality and ongoing transmission [5, 6].

Reliance on drug susceptibility testing (DST) using sputum culture in centralized reference laboratories limits access and results in delayed treatment. In 2013, less than 10% of bacteriologically confirmed TB cases were tested for MDR-TB globally [4]. The Xpert MTB/RIF test (Cepheid, Sunnyvale, CA) has the potential to both increase MDR-TB case detection and reduce time to treatment (TTT) initiation, through diagnosis of rifampicin resistance simultaneously with TB diagnosis [7]. Several countries, including South Africa, have now committed to expanding access to DST through Xpert, and the World Health Organization now supports the concept of universal access to DST [8].

However, improving diagnosis is insufficient in itself; health systems require capacity to treat the increasing numbers of patients diagnosed with rifampicin-resistant TB (RR-TB). With the implementation of Xpert in late 2011, South Africa has improved RR-TB case detection dramatically and diagnosed 26 023 cases in 2013, compared with 10 085 in 2011. The proportion of cases initiating treatment remains low; however, only 41% of diagnosed cases in 2013 are reported to have initiated second-line treatment [4]. Although routine recording of the number of cases starting treatment may be incomplete, the low rate of treatment uptake is supported by a small study in one province, where only 63% of diagnosed cases initiated treatment [6].

Reliance on centralized treatment in specialist hospitals is likely to contribute to long delays and lower numbers initiated on treatment, despite the introduction of more rapid diagnostics [9–12]. The introduction of genotypic DST for isoniazid and rifampicin using the rapid line probe assay ([LPA] Genotype MTBDR*plus*, Hain Lifesciences, Nehren, Germany) in place of conventional culture-based DST significantly decreased TTT in 2 rural areas of South Africa both served by centralized referral hospitals [13, 14]. However, the median TTT in both studies remained at 55 and 62 days with LPA, with significant delays reported between LPA results and treatment initiation. These data suggest that the greatest benefits will be seen when new diagnostics are introduced in the context of greater access to treatment through decentralized and community-based care. We aimed to assess the impact of Xpert implementation on the proportion of diagnosed cases initiating treatment and TTT initiation in the context of routine implementation of decentralized RR-TB care and management in Khayelitsha, a large periurban township in Cape Town.

## METHODS

### Setting

Khayelitsha township, with a population of approximately 400 000 [15], has one of the highest burdens of human immunodeficiency virus (HIV) and TB in the country and globally. In 2010, antenatal HIV prevalence was 33% and the TB case notification rate was at least 1500 per 100 000 per year [16, 17]. Rifampicin-resistant TB was found among 4.5% and 11.2% of new and previously treated TB cases, respectively [2].

A program, developed by Médecins sans Frontières in partnership with the City of Cape Town and the Western Cape Provincial Government, to decentralize RR-TB care and integrate management into existing primary care TB and HIV services was implemented in late 2007. Implementation was incremental, starting with extensive training of primary care clinic staff, TB infection control interventions, and introduction of clinic-based RR-TB registers. Additional inputs included the following: individual specific RR-TB counseling, social assistance and patient support groups, routine home visits and family education, contact tracing and screening, local audiometry screening, mentoring support for clinicians, initial strengthening of the standard RR-TB drug regimen, and a local subacute in-patient service. Patients were hospitalized only if they were clinically unstable and unable to attend their clinic daily for treatment (a requirement for ambulatory treatment). Treatment was provided through all 11 primary care facilities in the Khayelitsha subdistrict. This included 8 primary care clinics and 3 larger community health centres.

The full decentralized model of care has been described previously [18] along with patient and treatment outcomes [19]. It is estimated that the complete model of care was in place by the end of 2010, and the decentralized model has subsequently been implemented routinely across the City of Cape Town. Evaluation of the Khayelitsha RR-TB program was approved the University of Cape Town ethical review committee (Reference HREC 540/2010).

### Drug Susceptibility Testing

During 2007 and 2008, routine DST for rifampicin moved from conventional culture-based DST (liquid culture for isolation of *M tuberculosis* followed by susceptibility testing on solid media) to LPA, predominantly after a positive liquid culture (culture with LPA). Direct LPA testing of smear-positive patient samples (prior to culture) was performed uncommonly and only on specific clinician request, but this practice increased over subsequent years. Data on whether LPA was conducted from a cultured or direct specimen was not readily available. Line probe assay testing initially occurred in a study assessing the performance of the LPA in the central routine diagnostic laboratory in Cape Town [20]. Hence, over these 2 years, Khayelitsha patients were tested with either technique in a random manner. From January 2009, LPA became the standard for routine DST for isoniazid and rifampicin (also done in the central Cape Town laboratory).

Before October 2011, DST was available only for TB cases considered at high RR-TB risk: those not responding to first-line TB treatment, those previously treated for TB, those with close RR-TB contacts, healthcare workers, and those with mining or prison history. In late 2011, all individuals with presumptive TB were tested with the Xpert MTB/RIF test, which was conducted in a laboratory located at the district hospital in Khayelitsha. Although Xpert became the principal TB test, culture and LPA could also be requested routinely by clinic staff if considered appropriate (often for Xpert-negative patients). Therefore, not all RR-TB patients were diagnosed with Xpert. All RR-TB cases diagnosed with Xpert were confirmed using LPA on a second sputum sample submitted at the same time as the one undergoing Xpert.

### Data Collection and Analysis

The study included all patients diagnosed with RR-TB who were considered to be a resident in Khayelitsha subdistrict. Data from RR-TB patients diagnosed and treated before January 2008 were collated retrospectively through a medical record review. Due to incomplete data on all diagnosed cases in this time period, particularly those not starting treatment, the percentage of diagnosed cases started on treatment was not assessed. From January 2008, all data on diagnosed RR-TB cases residing in Khayelitsha were collected prospectively, through paper-based registers at clinic level (all 11 primary care facilities), with monthly data entry into an electronic database. Additional cases diagnosed outside of Khayelitsha (ie, at secondary and tertiary hospitals) were entered based on laboratory reports.

Data from January 2009 until the end of June 2013 were considered complete and were used to assess the proportion of diagnosed cases starting treatment. A minimum of 12 months follow-up time was allowed to assess treatment initiation.

Time to treatment initiation was determined as the time from collection of a sputum sample from which rifampicin resistance was determined to the date second-line anti-TB treatment was initiated (as recorded in both the register and the patient record). Patients with microbiologically unconfirmed RR-TB were excluded (predominantly close pediatric contacts of known cases), as were patients initiating a second treatment episode for RR-TB. Patients with discordant rifampicin resistance results by Xpert and LPA were also excluded (14 cases during 2012–2013). Data on percentage of diagnosed cases receiving treatment (at any point with a minimum of 12 months follow-up time) and TTT were assessed by year(s) to correlate with stage of decentralized program implementation and DST method.

Proportions were compared with χ^2^ analysis (*P* values mid-P exact, 2-tailed). Medians were compared with the Mann–Whitney *U* test, and changes in TTT over multiple time periods were assessed with the Jonckheere test. Kaplan-Meier analysis was used to show TTT, comparing the pre-Xpert time period (2010–2011) with the Xpert implementation period (2012–2013). The association of factors with TTT for patients diagnosed in 2012–2013 (Xpert as principal diagnostic test) was performed with multivariate Cox regression, with censoring at death for patients not started on treatment. Factors considered to be clinically and programmatically relevant to TTT were assessed in the multivariate analysis, regardless of significance on univariate analysis. These included the following: smear microscopy status, whether RR-TB was diagnosed with Xpert, HIV status, TB disease site (pulmonary versus extrapulmonary), age, and sex. All analyses were conducted with IBM SPSS Statistics (version 22).

## RESULTS

Based on the retrospective review, 158 eligible patients were started on RR-TB treatment before the decentralized program (2003–2006). During the first 2 years of the decentralized program (limited decentralization, 2007–2008), 257 cases were recorded prospectively as starting treatment (Table 1). Between January 2009 and June 2013 inclusive, 938 eligible patients were diagnosed with microbiologically confirmed RR-TB; 74% were infected with HIV, 49% were female, and the median age was 33 years (IQR, 26–40). Among these 938 patients, 817 (87%) were initiated on second-line treatment.

**Table 1.** Description of Principal Diagnostic Test in use and Model of Care Interventions, Number of RR-TB Patients Diagnosed and Treated and Median TTT, by Year(s) in Khayelitsha

	2003–2006 Culture-Based DST	2007–2008 Culture-Based DST	2007–2008 Culture With LPA	2009	2010	2011	2012	2013^a^
DST Method				LPA (After Culture or Direct From Specimen)			Xpert	
RR-TB model of care	Centralized	Limited decentralization		Improved program implementation		Full decentralization		
No. diagnosed	Not available			218	212	195	219	94
No. treated	158	95	162	182	182	173	191	89
% treated	Not available			83.5	85.8	88.7	87.2	94.7
Median TTT (days) (IQR)	71 (49–134)	76 (62–111)	50 (38–73)	40 (29–53)	34 (21–51)	28 (16–40)	13 (6–35)	8 (5–25)

Abbreviations: DST, drug susceptibility testing; IQR, interquartile range; LPA, line probe assay; RR-TB, rifampicin-resistant tuberculosis; TTT, time to treatment.

^a^ Half year only (January to June inclusive).

**Figure 1.**
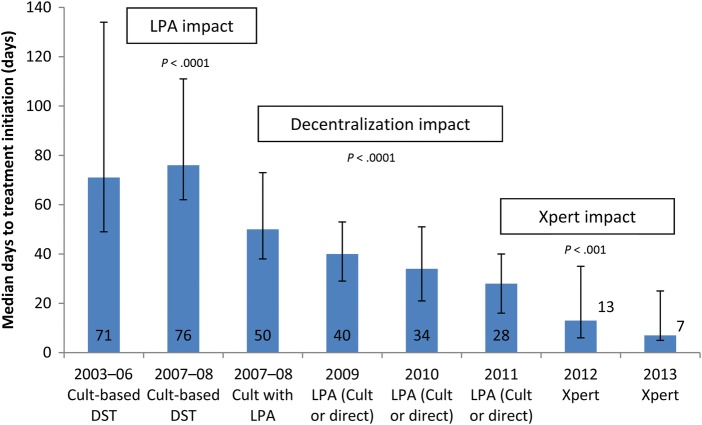
Median time to treatment (days) by year and diagnostic method (error bars represent interquartile range). Abbreviations: DST, drug susceptibility testing; LPA, line probe assay.

**Figure 2.**
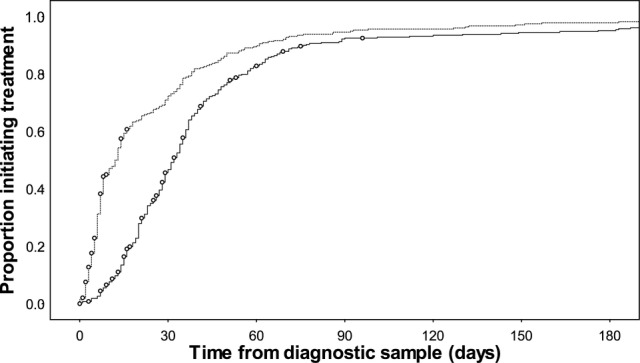
Time to treatment initiation comparing patients diagnosed in 2010–2011 with those diagnosed in 2012–2013 (Xpert implementation) (Kaplan–Meier, deaths before treatment censored [○]).

Median TTT was similar between patients treated during 2003–2006 and that subset of patients treated during 2007–2008 who were diagnosed with culture-based DST (*P* = .45) (Table 1 and Figure 1). Comparing patients diagnosed with culture-based DST and those diagnosed using culture with LPA during the same time period in 2007–2008, the use of LPA was associated with a significantly lower TTT (median 76 days) compared with culture-based DST (50 days; *P* < .0001). The impact of decentralization was assessed by comparing patients diagnosed by LPA only during 2007–2008 (LPA) and all patients from the subsequent 3 years; 2009, 2010, and 2011. During these 4 time periods, there was a significant decline in TTT (*P* < .0001, test for trend), resulting in a median TTT of 28 days in 2011 (Figure 1). By the end of 2010, implementation of the decentralized program was complete. Xpert was implemented at the end of 2011 and was associated with a further decline in TTT to a median of 8 days in 2013 (*P* < .0001, comparison of 2010–2011 with 2012–2013). Treatment initiation over the period of Xpert implementation (2012–2013), compared with the preceding 2-year period (2010–2011), with censoring for deaths before treatment, is shown in Figure 2.

The percentage of diagnosed patients initiating treatment was considered accurate from 2009 onwards, and it was consistently above 80% in this timeframe (Table 1). Although follow-up time was longer for patients diagnosed in earlier years, only 0.4% (3 of 728) of patients were initiated on treatment beyond 12 months for the years 2009–2012 inclusive. The introduction of Xpert (2012–2013) was not associated with an increase in the proportion initiating treatment compared with 2010–2011 (*P* = .36). The impact of Xpert implementation on percentage treated and TTT among patients infected with HIV was also assessed from 2009 onwards (Table 2). Between 2009 and 2013, HIV infection status was known for 97% (911 of 938) of diagnosed patients. Among those with unknown HIV status, treatment was initiated for 22% (6 of 27), with all 6 of these patients refusing HIV testing. Before Xpert implementation (2009–2011), 93.5% of HIV-negative patients initiated treatment compared with 85.5% among patients infected with HIV (*P* = .001). This difference remained significant in 2012–2013 with Xpert implementation; 100% of HIV-negative patients initiated treatment compared with 88.9% of those with HIV infection (*P* = .0002). Among the 24 HIV-infected patients who did not initiate treatment over this period, 12 (50%) are known to have died before treatment could be initiated. Among those starting treatment, there was no difference in median TTT by HIV status with either LPA or Xpert as the principal means of diagnosis (Table 2).

**Table 2.** Number of RR-TB Cases Diagnosed, Treated, and Median TTT by HIV Status and Year(s) in Khayelitsha

	2009	2010	2011	2012	2013^a^
DST Method	LPA (After Culture or Direct From Specimen)			Xpert	
HIV-infected					
No. diagnosed	165	154	135	146	71
No. treated	136	131	121	126	67
% treated	82.4	85.1	89.6	86.3	94.4
Median TTT (days) (IQR)	42 (30–57)	34 (22–52)	28 (16–42)	14 (6–35)	8 (5–29)
HIV-negative					
No. diagnosed	46	50	57	65	22
No. treated	42	49	52	65	22
% treated	91.3	98.0	91.2	100.0	100.0
Median TTT (days) (IQR)	34 (28–49)	34 (18–49)	25 (16–36)	12 (6–33)	7 (4–14)

Abbreviations: DST, drug susceptibility testing; HIV, human immunodeficiency virus; IQR, interquartile range; LPA, line probe assay; RR-TB, rifampicin-resistant tuberculosis; TTT, time to treatment.

^a^ Half year only (January to June).

During 2012–2013, with Xpert implementation, only 48% (150 of 313) of RR-TB cases were actually diagnosed using Xpert. The remainder were diagnosed predominantly with LPA after positive culture. Over this same time period (2012–2013, Xpert implementation), significant factors associated with improved TTT on univariate analysis were sputum smear-positive microscopy status, diagnosis by Xpert, compared with LPA and pulmonary TB. Both sputum smear positivity and diagnosis by Xpert (compared with LPA) remained significant on multivariate analysis, with adjusted hazard ratios of 1.65 and 2.29, respectively (Table 3). Sputum smear positivity remained significant in separate multivariate analyses among patients diagnosed with Xpert and those diagnosed with LPA (data not shown).

**Table 3.** Multivariate Cox Regression Analysis of Factors Associated With Treatment Initiation Over Time During the Xpert Implementation Period (*n* = 313, 2012–2013)^a^

Factor	N (%)	Univariate	Multivariate
		Hazard Ratio (95% Confidence Interval)	
Smear microscopy status			
Smear negative	129 (41%)	1.0 (reference)	1.0 (reference)
Smear positive	144 (46%)	1.60 (1.24–2.08)	1.65 (1.24–2.18)
Missing	40 (13%)	0.64 (0.43–0.95)	0.81 (0.54–1.23)
Xpert diagnosis			
No	163 (52%)	1.0 (reference)	1.0 (reference)
Yes	150 (48%)	2.46 (1.92–3.16)	2.29 (1.76–2.97)
HIV status			
HIV negative	87 (28%)	1.0 (reference)	1.0 (reference)
HIV infected	217 (69%)	0.80 (0.62–1.03)	1.08 (0.80–1.45)
Missing	9 (3%)	0.00	0.00
TB disease site			
Extrapulmonary	24 (8%)	1.0 (reference)	1.0 (reference)
Pulmonary/both	264 (84%)	1.56 (1.01–2.40)	1.03 (0.66–1.63)
Missing	24 (8%)	1.41 (0.60–3.32)	0.75 (0.31–1.81)
Sex			
Female	154 (49%)	1.0 (reference)	1.0 (reference)
Male	159 (51%)	1.04 (0.82–1.32)	1.00 (0.78–1.28)
Age			
0–19	22 (7%)	1.0 (reference)	1.0 (reference)
20–29	105 (34%)	0.92 (0.56–1.52)	0.84 (0.49–1.44)
30–39	105 (34%)	0.84 (0.51–1.38)	0.86 (0.49–1.49)
40+	81 (26%)	0.90 (0.54–1.50)	0.91 (0.52–1.59)

Abbreviations: HIV, human immunodeficiency virus; TB, tuberculosis.

^a^ Note that an increased hazard ratio represents improved treatment initiation over time.

## DISCUSSION

We have previously shown that implementation of a decentralized, community-based program for drug-resistant TB in a high HIV, TB, and RR-TB setting improved case detection and reduced the time between diagnosis and treatment initiation for RR- TB [19]. The median TTT decreased from more than 70 days before the program to 28 days with the decentralized program fully functional and utilizing the LPA as the principal diagnostic test. Implementation of the Xpert MTB/Rif test for all individuals with presumptive TB was associated with a further reduction in TTT to a median of 7 days in 2013.

However, TTT needs to be viewed in the context of the proportion of diagnosed cases that initiate treatment. Before decentralization, data collection was relatively poor, but, based on data from elsewhere, it is likely that a significant proportion of diagnosed cases were not started on treatment [6, 21]. Improved staff training, community awareness campaigns, patient follow-up, and counseling (components of community-based care) were associated with proportions of diagnosed cases starting treatment consistently above 80%. Given the already high level of treatment initiation due to decentralization of services, the slight increase that was seen with Xpert implementation was not statistically significant; however, this may be different in other settings with centralized RR-TB services.

Not unexpectedly, treatment initiation over time was significantly enhanced when patients were diagnosed using the rapid Xpert test during 2012–2013, compared with other diagnostic methods. Approximately half of all cases over this period were not diagnosed with Xpert, even though Xpert was the principal diagnostic test. The most common reason for this was Xpert-negative results and subsequent culture positivity. This finding suggests that, particularly in high HIV-prevalence settings such as this, a single Xpert test may not be sufficient for improved case detection and that more sensitive methods such as culture followed by LPA may still be required.

During the same Xpert implementation period (2012–2013), treatment initiation over time was also significantly enhanced for sputum smear-positive patients, compared with smear-negative patients. Both of these factors remained significant in multivariate analyses, suggesting independent effects. Sputum smear positivity is an indicator of infectiousness, and, from a public health perspective, starting these patients on treatment more rapidly represents significant potential to reduce community transmission, particularly given the rapid reduction in infectiousness with appropriate treatment recently demonstrated [3].

Given the high early mortality rate among RR-TB patients coinfected with HIV [5], Xpert may be particularly beneficial in this group. Although there were similar decreases in TTT between HIV-infected and uninfected patients with implementation of the decentralized program, and with Xpert, there was a consistent difference in the proportion of diagnosed patients initiating treatment with HIV status. Between 2012 and 2013, when Xpert was in place, 100% of HIV-negative patients initiated treatment compared with 89% of those with HIV. Among the HIV-infected patients not started on treatment, half are known to have died before treatment could be initiated, and these deaths occurred very rapidly. Given that these patients were not initiated on treatment, data on their immune and antiretroviral treatment (ART) status is largely unknown. However, it is likely that they were not receiving ART and consequently had low CD4 levels at the time of diagnosis, contributing to early mortality [22]. Although these data suggest that earlier diagnosis with a more sensitive, point of care test might be beneficial, earlier presentation for both ART and TB diagnosis is required to avert additional mortality among patients infected with HIV.

The reduction in TTT with the LPA in 2007 and 2008 confirms a previous study showing a similar, significant reduction with use of the LPA [13]. In that study, although TTT was reduced, significant delays were still observed, confirming the observation by Jacobson et al [13] that improvements in patient recall and health system infrastructure were required in order to see the greatest impacts from new diagnostics. In the absence of improved capacity to provide treatment, it is likely that the full benefits of new diagnostics will not be realized.

Although we have shown a significant reduction in TTT, the full impact of Xpert on TTT was probably not seen in the first year (2012) of implementation in Khayelitsha. Implementation of new diagnostics and new algorithms often take some time to become embedded into routine practice. In addition, there were initial concerns about lower positive predictive value in settings with relatively low proportions of rifampicin resistance [7]. Although it has recently been shown that positive-predictive value is high for Xpert in Cape Town [23], at the time of this study, there was no guidance or algorithm in place on how to manage these patients. Therefore, we excluded the relatively small number of patients where discordant results between Xpert and the LPA for rifampicin resistance were observed. Patients with discordant results are often referred for specialist consideration, which may result in delayed treatment initiation.

We assessed the impact of various interventions over several years. Because these interventions were not implemented into a static healthcare system, it is difficult to attribute direct cause and effect, particularly with multiple simultaneous interventions. In particular, it would be interesting to assess whether decentralization resulted in continued declines in TTT in the absence of Xpert. Without accurate data on the date that clinics actually received RR-TB results, it is hard to assess how much delay is associated with recalling the patient, counseling, and the treatment initiation process. However, given the median TTT of 28 days in 2011, it is likely that this process was reasonably streamlined at this point, with limited capacity for further reductions. Despite this limitation, these are real programmatic data confirming the benefits of both decentralization of RR-TB care and Xpert diagnosis for all individuals with presumptive TB. These findings are particularly relevant for South Africa, where access to Xpert has been scaled up but full access to decentralized, community-based care is not yet a reality.

The use of routine, programmatic data in this study may also be considered a limitation. Although data collected retrospectively, before 2009, are likely to be poor, data from 2009 onward were collected prospectively, with triangulation from several different sources and continuous data checking and feedback to ensure quality. Nonetheless, there may have been some patients diagnosed and never started on treatment who were missed entirely.

We described TTT initiation from the date that a specimen was given from which RR-TB was detected. Given that before 2011 not all TB cases were offered DST routinely, it is likely that some patients had significant delays from first TB diagnosis to RR-TB diagnosis, and we have not assessed these delays. However, this omission is likely to enhance any impact of Xpert, which includes not just rapid testing but also the potential to offer DST to all TB cases.

## CONCLUSIONS

Simultaneous improvements in case detection and access to treatment are required in order to avert mortality and reduce ongoing transmission. The World Health Organization estimates that approximately half a million new cases of MDR-TB arise annually [4]. In 2013, only 28% of these cases were diagnosed, and only 71% of these were started on appropriate treatment, most likely with considerable delays. Xpert has the potential to increase case detection dramatically, principally through the opportunity to provide universal drug susceptibility testing in high TB-burden settings. Xpert also affords the opportunity to initiate appropriate second-line treatment rapidly, offering potential to not only improve patient outcomes but also to reduce community transmission.
